# Apelin Enhances the Effects of *Fusobacterium nucleatum* on Periodontal Ligament Cells In Vitro

**DOI:** 10.3390/ijms24054733

**Published:** 2023-03-01

**Authors:** Pablo Cores Ziskoven, Andressa V. B. Nogueira, Lorena S. Gutierrez, Jens Weusmann, Sigrun Eick, Nurcan Buduneli, James Deschner

**Affiliations:** 1 Department of Periodontology and Operative Dentistry, University Medical Center of the Johannes Gutenberg University, 55131 Mainz, Germany; 2 Department of Diagnosis and Surgery, School of Dentistry at Araraquara, São Paulo State University-UNESP, Araraquara 14801-385, SP, Brazil; 3 Laboratory of Oral Microbiology, Department of Periodontology, University of Bern, 3010 Bern, Switzerland; 4 Department of Periodontology, School of Dentistry, Ege University, 35040 Izmir, Turkey

**Keywords:** apelin, APJ, periodontal ligament cells, *Fusobacterium nucleatum*, periodontitis, obesity

## Abstract

This study aimed to explore effects of *Fusobacterium nucleatum* with or without apelin on periodontal ligament (PDL) cells to better understand pathomechanistic links between periodontitis and obesity. First, the actions of *F. nucleatum* on COX2, CCL2, and MMP1 expressions were assessed. Subsequently, PDL cells were incubated with *F. nucleatum* in the presence and absence of apelin to study the modulatory effects of this adipokine on molecules related to inflammation and hard and soft tissue turnover. Regulation of apelin and its receptor (APJ) by *F. nucleatum* was also studied. *F. nucleatum* resulted in elevated COX2, CCL2, and MMP1 expressions in a dose- and time-dependent manner. Combination of *F. nucleatum* and apelin led to the highest (*p* < 0.05) expression levels of COX2, CCL2, CXCL8, TNF-α, and MMP1 at 48 h. The effects of *F. nucleatum* and/or apelin on CCL2 and MMP1 were MEK1/2- and partially NF-κB-dependent. The combined effects of *F. nucleatum* and apelin on CCL2 and MMP1 were also observed at protein level. Moreover, *F. nucleatum* downregulated (*p* < 0.05) the apelin and APJ expressions. In conclusion, obesity could contribute to periodontitis through apelin. The local production of apelin/APJ in PDL cells also suggests a role of these molecules in the pathogenesis of periodontitis.

## 1. Introduction

Periodontitis is a chronic inflammatory disease mainly caused by a subgingival dysbiotic microbiota whose balance is shifted by several factors [1]. Additionally, there is also a dysbiotic status between the host and the subgingival microbiota in periodontitis. Hyperinflammatory immune responses of the host to this microbiota can lead to alveolar bone resorption and eventually tooth loss [1]. Risk factors such as smoking or genetic predisposition can contribute to the initiation and progression of periodontitis [2]. There is strong evidence that periodontitis is associated with systemic diseases and conditions, such as diabetes mellitus, cardiovascular disease, hypertension, obesity, and metabolic syndrome. It is thought that the oral microorganisms, their components, or metabolites as well as inflammatory mediators get into the systemic circulation and therefore to other parts of the human body [3,4,5,6,7,8]. Obesity is defined as abnormal or excessive fat accumulation that presents a risk to health [9]. Because adipose tissue is not only an energy reservoir but also a metabolic organ, dysregulation of cytokines, hormones, and metabolites occurs when this tissue increases [10]. There is evidence that obese individuals have systemically high levels of CRP, TNF-α, and IL-6 in comparison to normal-weight subjects and, therefore, are in a chronic subclinical inflammatory state [11]. A lot of possible pathomechanisms have been suggested to be responsible for the link between periodontitis and obesity, such as adipokines [12]. Adipokines are cytokines produced by adipocytes, but also by other cell types, such as periodontal cells [13,14,15,16,17,18]. Various adipokines such as leptin, visfatin, adiponectin, and resistin have been identified and studied in regard to systemic diseases. It is suggested that these adipokines have a wide range of functions, which include regulation of insulin metabolism, thirst and hunger sensation, angiogenesis, energy balance, bone metabolism, coagulation, and hematopoiesis, as well as inflammation and its resolution [13,19]. Adiponectin has mainly anti-inflammatory effects, whereas resistin, visfatin, and leptin are more pro-inflammatory [20,21]. Another adipokine, which has been rather less studied so far, is apelin. Apelin was first isolated and described in 1998 [22]. As early as 1993, the apelin receptor (angiotensin II protein J receptor, APJ) had been discovered in humans as a G protein-coupled receptor whose gene locus is located on chromosome 11 [23]. Apelin has a wide range of effects, which differ depending on cell types and tissues. Originally, apelin was isolated from tissues of the central nervous system. Accordingly, the molecule was found to be important in central signal transduction [24]. As research progressed, the apelin-APJ system was discovered in other tissues as well. For example, the molecule interferes with the regulation of bone turnover by modulating apoptosis, proliferation, and differentiation of osteoblasts [25,26]. It has been shown that apelin levels are increased in systemic diseases and conditions such as obesity and diabetes [27,28]. A recent study looked at serum levels of apelin in diabetes and/or periodontitis patients [29]. Those patients who suffered from both diabetes and periodontitis exhibited the highest serum levels of apelin as compared to healthy individuals. Another study could show that the salivary apelin levels of diabetic patients with periodontitis were increased as compared to healthy individuals [30]. This adipokine also has modulatory properties regarding inflammation. For example, apelin can increase the expression of TNF-α and IL-1β in glial cells, but at the same time downregulate inflammatory mediators in lung and heart cells [31,32]. Therefore, apelin could be a critical molecule, which may mediate the harmful effects of obesity on periodontal tissues. The aim of this in vitro study was to explore the regulatory effects of *Fusobacterium nucleatum* in the presence or absence of apelin on periodontal ligament (PDL) cells in order to test the hypothesis that apelin might be one of the pathomechanistic links between periodontal disease and obesity.

## 2. Results

### 2.1. Regulation of COX2, CCL2, and MMP1 Expressions by F. nucleatum

**Figure 1:**
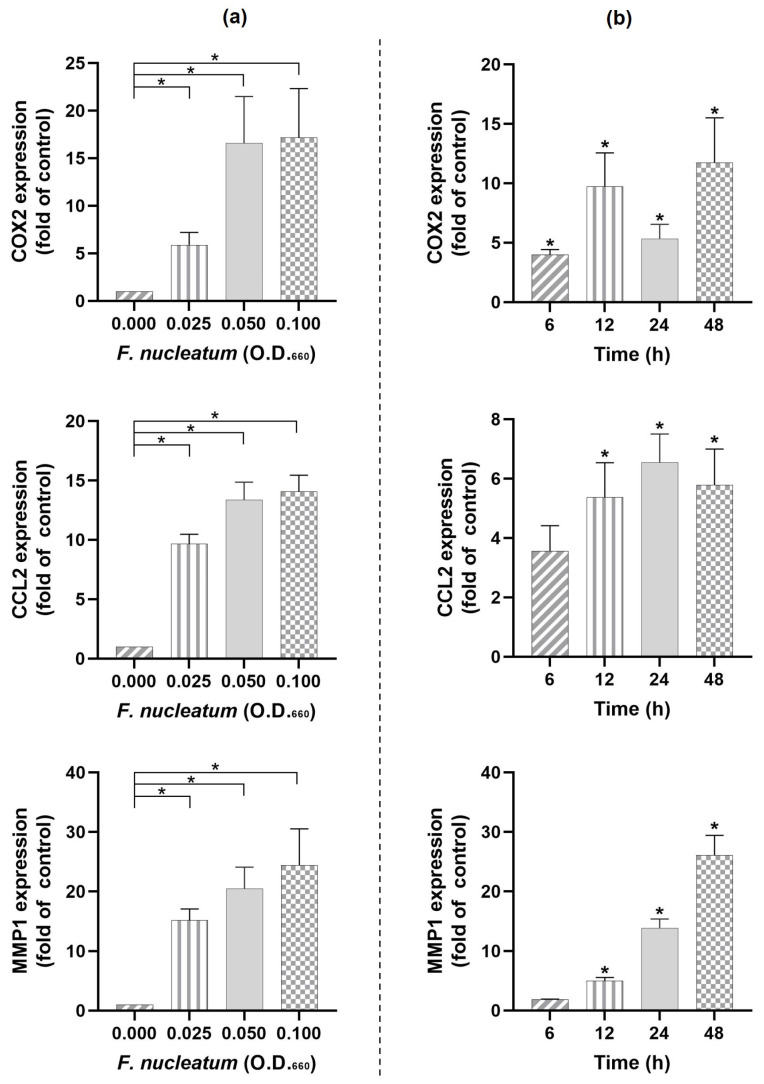
(**a**) Expressions of COX2, CCL2, and MMP1 in the presence and absence of different concentrations of *F. nucleatum* (O.D._660_: 0.000, 0.025, 0.050, and 0.100) in PDL cells at 24 h. (**b**) Expressions of COX2, CCL2, and MMP1 in the presence and absence of *F. nucleatum* (O.D._660_: 0.025) in PDL cells at different time points (6 h, 12 h, 24 h, and 48 h). Graphs show mean values and standard errors of the mean (SEM). * significantly (*p* < 0.05) different from unstimulated control.

First, we wanted to verify whether *F. nucleatum* would regulate the expression of COX2, CCL2, and MMP1 in PDL cells. *F. nucleatum* caused a significant (*p* < 0.05) and dose-dependent (O.D._660_: 0.000, 0.025, 0.050, and 0.100) upregulation of the pro-inflammatory and proteolytic molecules COX2, CCL2, and MMP1 with the highest expression for the highest bacterial concentration (O.D._660_ = 0.100) at 24 h (Figure 1a). In addition, the stimulatory effect of *F. nucleatum* (O.D._660_ = 0.025) on these molecules was also time-dependent (*p* < 0.05), as shown in Figure 1b.

### 2.2. Modulatory Effects of Apelin on Pro-Inflammatory Actions by F. nucleatum

**Figure 2:**
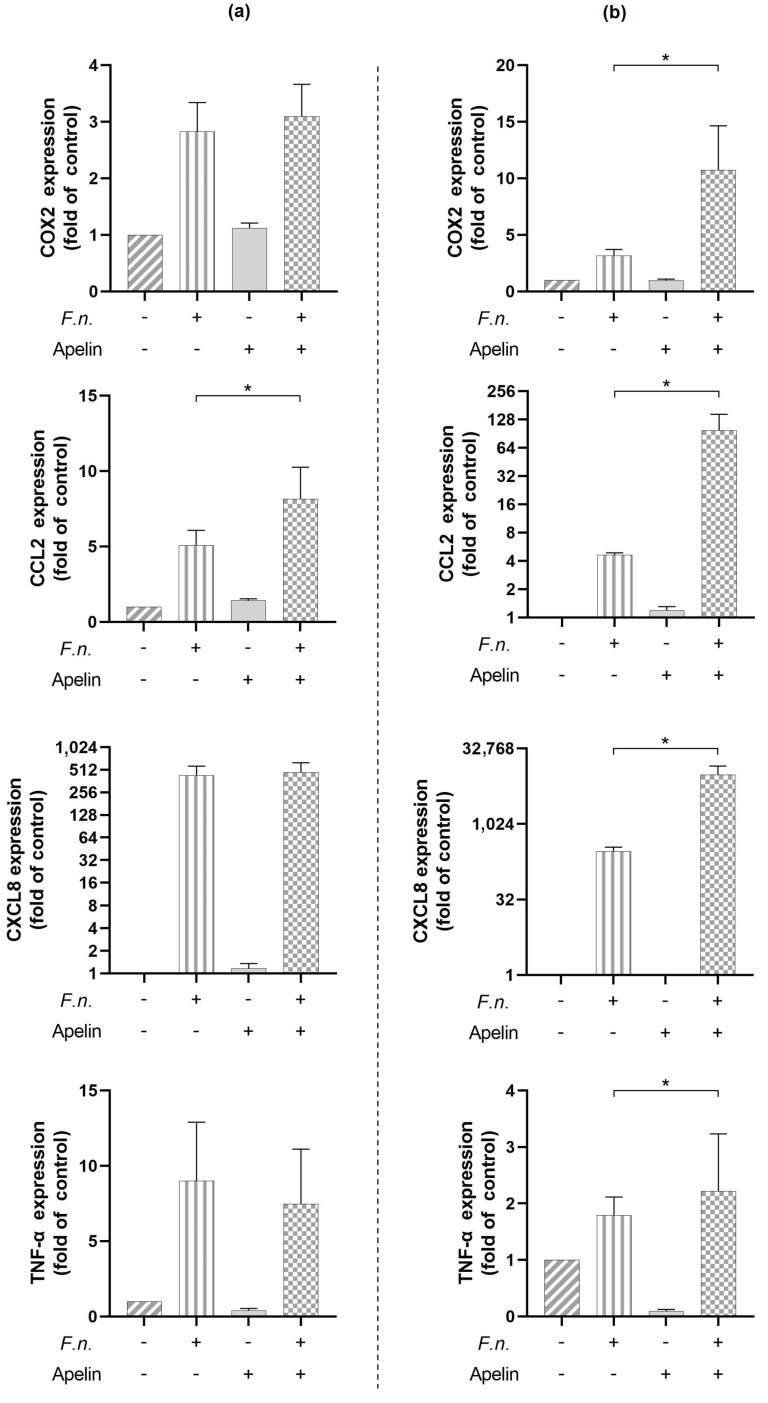
Effect of *F. nucleatum* (O.D._660_: 0.025) and/or apelin (1 ng/mL) on the expression of pro-inflammatory mediators (COX2, CCL2, CXCL-8, and TNF-α) at 24 h (**a**) and 48 h (**b**). Graphs show mean values and standard errors of the mean (SEM). * significant (*p* < 0.05) difference between cells exposed to *F. nucleatum* with and without apelin.

Next, we studied whether apelin (1 ng/mL) could modulate the stimulatory actions of *F. nucleatum* (O.D._660_ = 0.025) on the expression of pro-inflammatory markers in PDL cells. Apelin was used at a concentration corresponding to physiological plasma levels and consistent with previous in vitro studies. For *F. nucleatum*, O.D._660_ = 0.025 was chosen because even this minimal dose had a proinflammatory effect on PDL cells, as evidenced by a significant increase in the expression of COX2, CCL2, and MMP1. As shown by real-time PCR analysis, apelin significantly (*p* < 0.05) increased the *F. nucleatum*-stimulated expression of CCL2 at 24 h (Figure 2a). For COX2, CXCL-8, and TNF-α, no significant modulatory effect of apelin on the *F. nucleatum*-triggered expression was observed at this time point (Figure 2a). Moreover, apelin caused a further significant (*p* < 0.05) elevation of the *F. nucleatum*-induced expressions of COX2, CCL2, CXCL-8, and TNF-α at 48 h (Figure 2b). This shows that the stimulatory influence of apelin on the effects of *F. nucleatum* was stronger at 48 h as compared to 24 h.

### 2.3. Modulatory Effects of Apelin on Markers Involved in Soft and Hard Tissue Turnover

**Figure 3:**
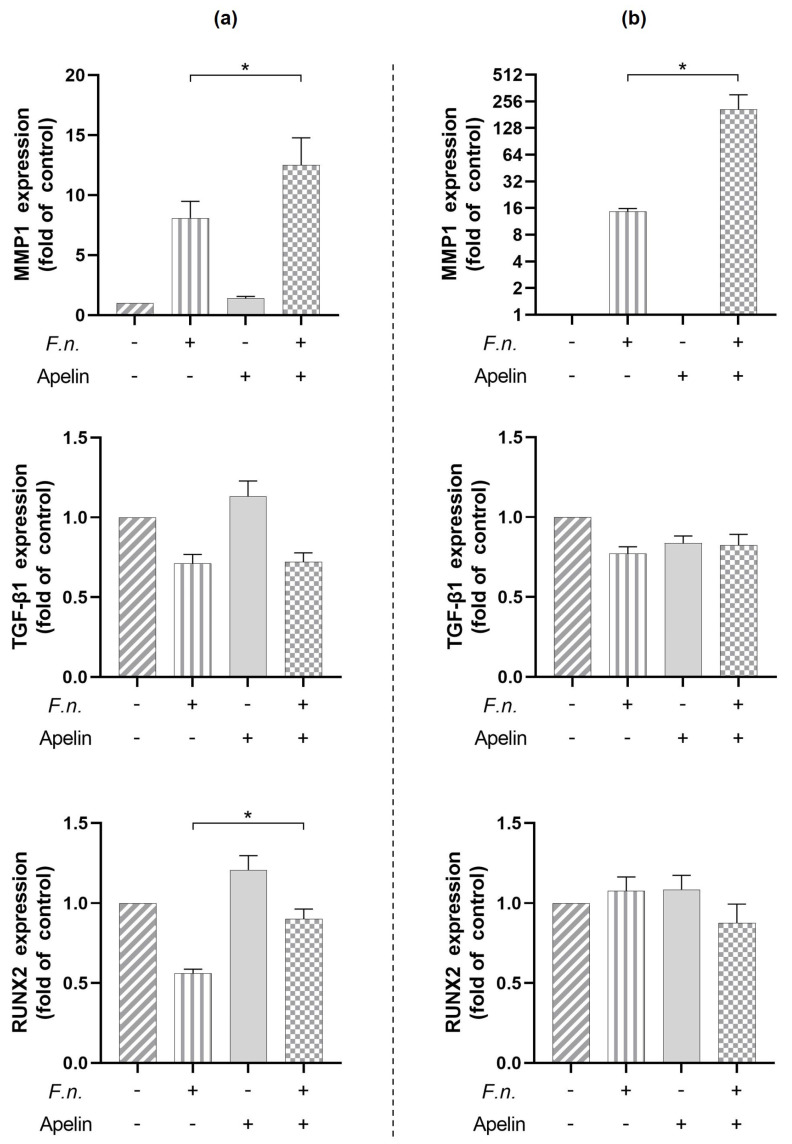
Effect of *F. nucleatum* (O.D._660_: 0.025) and/or apelin (1 ng/mL) on the expression of mediators involved in soft and hard tissue turnover (MMP1, TGF-β1, and RUNX) at 24 h (**a**) and 48 h (**b**). Graphs show mean values and standard errors of the mean (SEM). * significant (*p* < 0.05) difference between cells exposed to *F. nucleatum* with and without apelin.

We then examined the effect of apelin (1 ng/mL) on the regulation of MMP1, TGF-β1, and RUNX2 by *F. nucleatum* (O.D._660_ = 0.025) in PDL cells (Figure 3). *F. nucleatum* increased the expression of MMP1 at 24 h (Figure 3a) and 48 h (Figure 3b), and this upregulation was significantly (*p* < 0.05) enhanced by apelin at both time points. No upregulation by *F. nucleatum* was observed for TGF-β1 and RUNX2 at 24 h (Figure 3a) and 48 h (Figure 3b). Apelin had no significant effect on the actions of *F. nucleatum* on TGF-β1 at 24 h (Figure 3a) and 48 h (Figure 3b) and RUNX2 at 48 h (Figure 3b). Interestingly, apelin significantly (*p* < 0.05) counteracted the inhibitory effect of *F. nucleatum* on RUNX2 expression at 24 h (Figure 3a).

### 2.4. Involvement of Signaling Pathways in the Modulatory Effects of F. nucleatum and/or Apelin on CCL2 and MMP1 Expressions

**Figure 4:**
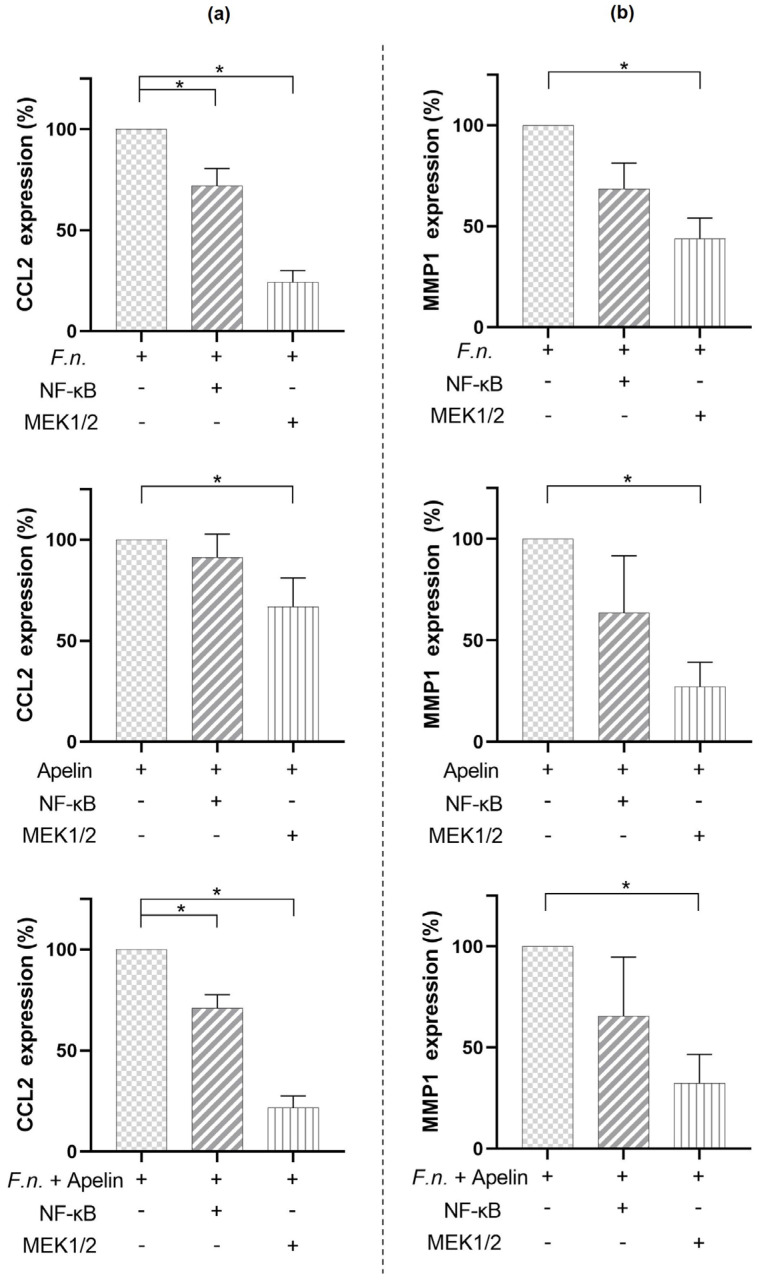
Involvement of signaling pathways in the effects of *F. nucleatum* (O.D._660_: 0.025) and/or apelin (1 ng/mL) on CCL2 (**a**) and MMP1 (**b**) expressions in PDL cells at 24 h. Cells were pre-incubated with specific inhibitors against NF-κB (PDTC, 10 µM) and MEK1/2 (U0126, 10 µM). Graphs show mean values and standard errors of the mean (SEM). * significant (*p* < 0.05) difference between groups.

We next sought to identify intracellular signaling pathways potentially involved in the actions of *F. nucleatum* on CCL2 and MMP1 in PDL cells. For this purpose, cells were pre-incubated with specific inhibitors for NF-κB or MEK1/2 signaling and subsequently stimulated with *F. nucleatum* (O.D._660_ = 0.025) and/or apelin (1 ng/mL). Pre-incubation of cells with an NF-κB inhibitor resulted in a significant (*p* < 0.05) downregulation of the CCL2 expression in cells treated with either *F. nucleatum* alone or in combination with apelin at 24 h (Figure 4a). In contrast, the expressions of CCL2 and MMP1 induced by *F. nucleatum* and/or apelin were always significantly (*p* < 0.05) inhibited by the MEK1/2 inhibitor after 24 h (Figure 4a,b). 

### 2.5. Effects of F. nucleatum on Apelin and Its Receptor 

**Figure 5:**
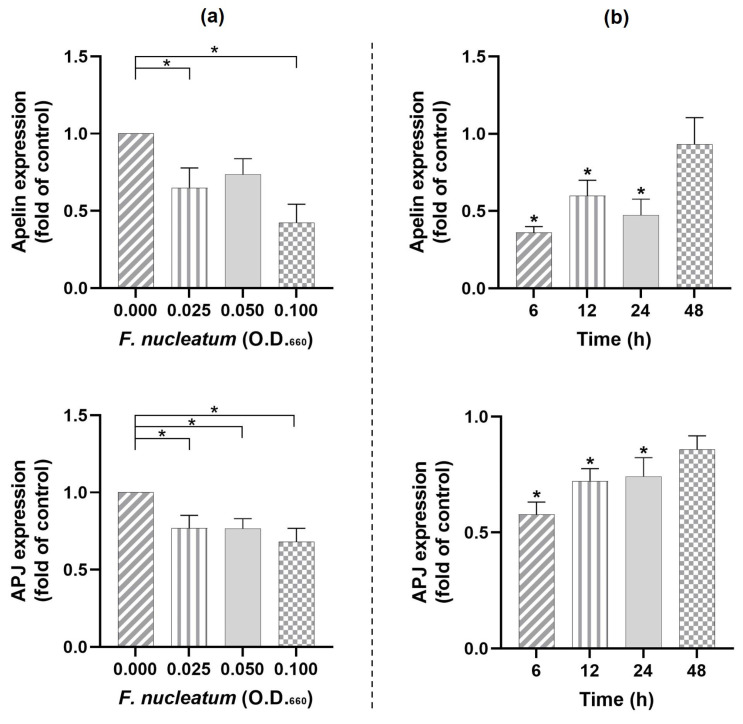
(**a**) Expressions of apelin and APJ in the presence and absence of different concentrations of *F. nucleatum* (O.D._660_: 0.000, 0.025, 0.050, and 0.100) in PDL cells at 24 h. (**b**) Expressions of apelin and APJ in the presence and absence of *F. nucleatum* (O.D._660_: 0.025) in PDL cells at different time points (6 h, 12 h, 24 h, and 48 h). Graphs show mean values and standard errors of the mean (SEM). * significantly (*p* < 0.05) different from unstimulated control.

We also investigated whether apelin is expressed in PDL cells and, if so, whether this adipokine as well as its receptor are regulated by *F. nucleatum* (O.D._660_ = 0.025). The periodontopathogen downregulated (*p* < 0.05) the expression of apelin and APJ over a variety of doses (Figure 5a). A slight time dependence was observed (Figure 5b).

### 2.6. Modulatory Effects of Apelin on CCL2 and MMP1 Protein Induced by F. nucleatum

**Figure 6:**
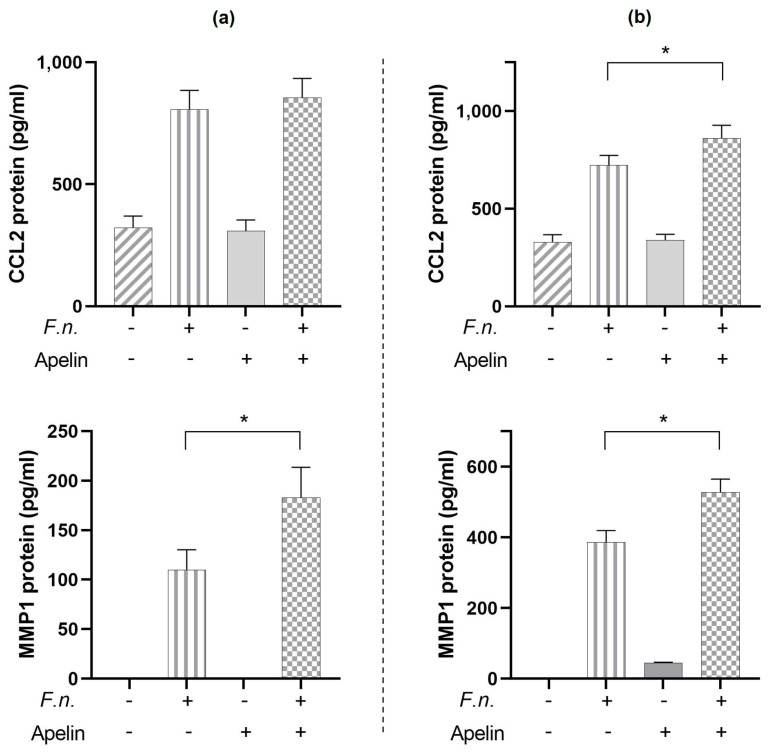
Protein levels of CCL2 and MMP1 in supernatants of PDL cells stimulated with *F. nucleatum* (O.D._660_: 0.025) and/or apelin (1 ng/mL) at 24 h (**a**) and 48 h (**b**). Graphs show mean values and standard errors of the mean (SEM). * significant (*p* < 0.05) difference between cells exposed to *F. nucleatum* with and without apelin.

Finally, we investigated whether apelin (1 ng/mL) can modulate the stimulatory effect of *F. nucleatum* (O.D._660_ = 0.025) on pro-inflammatory markers also at protein level in PDL cells. As detected by ELISA, *F. nucleatum* resulted in increased protein levels of CCL2 and MMP1 in cell supernatants at 24 h and 48 h (Figure 6a,b). Incubation of *F. nucleatum*-stimulated cells with apelin resulted in a further significant (*p* < 0.05) increase in protein levels of CCL2 at 48 h (Figure 6a) and of MMP1 at 24 h and 48 h (Figure 6b).

## 3. Discussion

This study aimed to investigate the modulatory effect of the adipokine apelin on the action of the periodontopathogen *F. nucleatum* on PDL cells to better understand the relationship between periodontitis and obesity. Interestingly, apelin was able to modify bacterial regulation of molecules related to inflammation and hard and soft tissue turnover. The combination of *F. nucleatum* and apelin resulted in the highest expression levels of pro-inflammatory and proteolytic molecules, suggesting that apelin may be a pathomechanistic link mediating deleterious effects of obesity on periodontal tissues. In addition, *F. nucleatum* caused downregulation of the expression of apelin and its receptor, suggesting a role of these molecules in the pathogenesis of periodontitis.

There is strong evidence for an association between periodontitis and obesity [12,33]. It has been shown in several studies of our research group that adipokines represent a possible pathomechanistic link underlying the association between periodontitis and obesity [15,16,17,18,34,35,36,37]. Leptin, visfatin, and resistin exert pro-inflammatory effects on periodontal cells and tissues, whereas adiponectin has rather protective effects on periodontal cells [33]. However, with respect to the periodontium, almost nothing is known about the production, regulation, and action of apelin, another adipokine whose serum levels are altered in obesity [28]. Recently, Hirani et al. investigated the serum level of apelin in periodontally and systemically healthy individuals and periodontitis patients with and without type 2 diabetes [29]. The study showed that apelin levels were higher in the periodontitis group compared with the healthy control. When patients had concomitant periodontitis and obesity, apelin levels were highest. The authors concluded that the increased expression of apelin in patients with periodontitis and type 2 diabetes might indicate a possible role of this adipokine in inflammation and glucose regulation. Sarhat et al. examined the salivary apelin levels of periodontally diseased diabetic patients and of periodontally and systemically healthy individuals [30]. They also found the highest apelin levels in periodontitis patients with diabetes. In our study, the periodontopathogen *F. nucleatum* led to a dose- and time-dependent upregulation of pro-inflammatory and proteolytic molecules. Interestingly, apelin caused an increase in the *F. nucleatum*-stimulated expression of these pro-inflammatory and proteolytic molecules. In this respect, our in vitro data confirm that apelin may be associated with inflammation. Lee et al. also investigated the relationship between apelin and periodontitis and found a decrease in apelin expression in gingival tissues from periodontitis patients, which is in contrast to the aforementioned studies [38]. Moreover, overexpression of apelin or treatment with exogenous apelin suppressed TNF-α-stimulated gene expressions of MMP1, IL-6, and COX2 in PDL cells [38]. Further studies are needed to clarify whether apelin levels in gingiva, sulcus fluid, saliva, and serum are increased or decreased in gingivitis and periodontitis, and whether apelin exerts pro- or anti-inflammatory effects. In addition, it should be investigated whether periodontal therapy results in a change in these apelin levels.

Furthermore, we were interested in whether apelin and its receptor are expressed in periodontal cells, and if so, whether this expression can be regulated by *F. nucleatum*. Our in vitro experiments with PDL cells showed that both apelin and its receptor are constitutively produced in these cells. Moreover, our experiments revealed that the periodontopathogen *F. nucleatum* inhibited the expression of apelin and its receptor. In the study by Lee et al., incubation of PDL cells and gingival fibroblasts with the inflammatory mediator TNF-α also resulted in downregulation of apelin [38]. Therefore, this and our study suggest that the apelin-APJ system is downregulated during periodontal infection and inflammation, at least initially. Because our results suggest that apelin exerts rather pro-inflammatory effects, the initial downregulation of apelin and its receptor may represent the host tissues’ attempt to limit inflammation and associated tissue destruction. However, our experiments also showed that this possibly tissue-protective downregulation of apelin and its receptor was no longer observed after 48 h, which may suggest that in persistent periodontal infection, the apelin-APJ system may be of critical importance in the pathogenesis of periodontitis.

*F. nucleatum* is an obligate anaerobic gram-negative bacterium very prevalent in the subgingival biofilm and associated with the etiopathogenesis of periodontitis [39,40]. Infection with *F. nucleatum* alone has shown to cause alveolar bone loss in a murine experimental periodontitis [41]. When in combination with *T. forsythia* or *P. gingivalis*, *F. nucleatum* synergistically stimulated the host immune response and induced alveolar bone loss in this experimental periodontitis model [42,43]. *F. nucleatum*, such as other red complex bacteria, is associated with periodontitis [44]. As expected according to our previous studies [45,46,47], *F. nucleatum* led to increased expressions of pro-inflammatory and proteolytic molecules, underlining the special role of this bacterium in periodontal inflammation and destruction. As in our previous studies, *F. nucleatum* was used as lysate, so several factors may have been responsible for the observed stimulatory effects of *F. nucleatum*. Studies using live *F. nucleatum* or even biofilms consisting of a variety of different bacteria should be performed in the future to confirm the results of this study.

Our study clearly demonstrates that apelin can exert pro-inflammatory effects and thus enhance periodontal inflammatory processes. Although there are numerous publications on anti-inflammatory and thus protective effects of apelin [48,49], there are also studies that have demonstrated pro-inflammatory effects of apelin [32,50]. 

Our analyses regarding intracellular signal transductions suggest that pro-inflammatory effects of *F. nucleatum* and/or apelin are realized at least partially through MAPK and NF-kB. Further studies should clarify which other intracellular signaling pathways apelin uses for its modulatory effects. Our results are thus in agreement with other studies that have also shown that apelin uses the MAPK and NF-kB signaling pathways, among others, for its effects [27,48,51,52].

In the present study, apelin and APJ were also shown to be produced in periodontal cells and regulated by periodontal pathogenic bacteria, suggesting that apelin and APJ may play an important role in the pathogenesis of periodontitis. Interestingly, *F. nucleatum* led to downregulation of apelin and its receptor in PDL cells, which would imply an anti-inflammatory effect in accordance with the other results of this study. However, because the inhibitory effect of *F. nucleatum* was lost with increasing duration of bacterial incubation, this protective effect might also be lacking in persistent periodontal infection. Future studies should also address the apelin-APJ system in other cells of the periodontium, e.g., gingival epithelial cells, and fibroblasts.

The increased production of apelin by periodontal cells after bacterial stimulation suggests that this adipokine is increased in saliva, sulcus fluid, gingiva, and serum during periodontal inflammation. Clinical studies of experimental gingivitis and periodontitis as well as periodontal therapy, i.e., intervention, should further clarify the role of apelin locally in the periodontium but also systemically for the whole organism.

In summary, within its limitations, our in vitro study demonstrated that the adipokine apelin is able to modulate the effects of *F. nucleatum* on molecules associated with inflammation and hard and soft tissue turnover. Apelin was able to further increase the expression of pro-inflammatory and proteolytic molecules induced by *F. nucleatum*, which may suggest that apelin may be a pathomechanistic link mediating the deleterious effects of obesity on periodontal tissues. In addition, our study revealed that PDL cells express apelin and APJ and that these expressions are inhibited by *F. nucleatum*, suggesting a possible role for this adipokine and its receptor in the pathogenesis of periodontitis.

## 4. Materials and Methods

### 4.1. Cell Culture

A human PDL cell line PDL26 was used for cell culture. As described previously, this cell line was obtained from a third molar tooth of a healthy, 26-year-old non-smoking patient [47]. Cells were first cultured in cell culture flasks provided with nutrient medium. The culture medium was Dulbecco’s Modified Eagle Medium (DMEM) GlutaMAX (Invitrogen, Karlsruhe, Germany) supplemented with 10% fetal bovine serum (FBS, Invitrogen). Furthermore, 100 units of penicillin and 100 μg/mL streptomycin (Invitrogen) were added to the medium. Cells were maintained in the incubator at 37 °C and with a humidified atmosphere of 5% CO_2_. Cells were cultured (1 × 10^5^ cells/well) on 6-well culture plates and grown until 70–80% confluence. The medium was changed every other day and 24 h before stimulation; the FBS concentration was reduced to 1%. The periodontopathogenic bacterium *F. nucleatum* ATCC 25586 was used at different concentrations (optical density, O.D._660_ = 0.025, 0.050, and 0.100) to simulate microbial infection in vitro. The bacterial strain was pre-cultivated on Schaedler agar plates (Oxoid, Basingstoke, UK) in an anaerobic atmosphere for 48 h. Successively, bacteria were suspended in phosphate-buffered saline (O.D._660_ = 1, corresponding to 1.2 × 10^9^ bacterial cells/mL) and submitted twice to ultrasonication (160 W for 15 min) leading to total killing. Furthermore, apelin (recombinant human apelin protein, Abcam, Cambridge, United Kingdom) was used for in vitro stimulation at a concentration corresponding to physiological plasma levels (1 ng/mL) and consistent with previous in vitro studies [53,54,55]. In addition, cells were pre-incubated with PDTC (10 µM, Cell Signaling Technology, Danvers, MA, USA), a specific inhibitor of NF-κB, and U0126 (10 µM, Calbiochem, San Diego, CA, USA), a specific inhibitor of MEK1/2 signaling. Untreated cells served as control. 

### 4.2. Real-Time PCR

RNA isolation was performed using RNeasy Mini Kit (Qiagen, Hilden, Germany) according to the manufacturer’s instructions. To determine the RNA concentration, the spectrophotometer NanoDrop ND-2000 (Thermo Fischer Scientific, Waltham, MA, USA) was used. Five hundred ng of total RNA was reverse transcribed using iScrip Select cDNA Synthesis Kit (Bio-Rad Laboratories, Munich, Germany) according to manufacturer’s protocol. Gene expression analysis of apelin and its receptor (APJ), C-C motif chemokine ligand 2 (CCL2), cyclooxygenase-2 (COX-2), C-X-C motif chemokine ligand 8 (CXCL8), glyceraldehyde-3-phosphate dehydrogenase (GAPDH), matrix metalloproteinase 1 (MMP1), runt-related transcription factor 2 (RUNX2), transforming growth factor-beta 1 (TGF-β1), and tumor necrosis factor alpha (TNF-α), was performed by real-time PCR using the PCR thermal cycler CFX96 (Bio-Rad Laboratories), SYBR green PCR master mix (QuantiFast SYBR Green PCR Kit, Qiagen), and specific primers (QuantiTect Primer Assay, Qiagen). One µL of cDNA was mixed with 12.5 µL master mix, 2.5 µL primer, and 9 µL nuclease-free water. The mix was heated at 95 °C for 5 min, followed by 40 cycles of denaturation at 95 °C for 10 s, and a combined annealing/extension step at 60 °C for 30 s. Data were analyzed by comparative threshold cycle method. 

### 4.3. ELISA

The protein levels of CCL2 and MMP1 in the cell supernatants were measured using commercially available ELISA kits (DuoSet, R&D Systems, Minneapolis, MN, USA) according to the manufacturer’s instructions. The optical density was determined using a microplate reader (BioTek Instruments, Winooski, VT, USA) set to 450 nm. The readings at 450 nm were subtracted from the readings at 540 nm for optical correction as per manufacturer’s recommendation. Cell numbers were checked and there was no significant difference between groups.

### 4.4. Statistical Analysis

The statistical analysis was performed using the software GraphPad Prism (version 9.2.0, GraphPad Software, San Diego, CA, USA). For data analysis, mean values and standard errors of the mean (SEM) were calculated. Data were checked for normal distribution and, subsequently, analyzed with the *t*-test (parametric) or Mann–Whitney-U test (non-parametric). For multiple comparisons, ANOVA or the Kruskall–Wallis test was applied, depending on normal distribution. The Dunnet’s (parametric) or Dunn’s test (non-parametric) served as post hoc tests. The significance level was set at *p* < 0.05 for all experiments. 

## Data Availability

Data sharing is not applicable to this article.
